# In Vitro and In Vivo Anti-*Candida* Activity and Structural Analysis of Killer Peptide (KP)-Derivatives

**DOI:** 10.3390/jof7020129

**Published:** 2021-02-10

**Authors:** Tecla Ciociola, Thelma A. Pertinhez, Tiziano De Simone, Walter Magliani, Elena Ferrari, Silvana Belletti, Tiziana D’Adda, Stefania Conti, Laura Giovati

**Affiliations:** 1 Department of Medicine and Surgery, University of Parma, 43126 Parma, Italy; tecla.ciociola@unipr.it (T.C.); thelma.pertinhez@unipr.it (T.A.P.); tiziano.desimone@unipr.it (T.D.S.); walter.magliani@unipr.it (W.M.); elena.ferrari@unipr.it (E.F.); silvana.belletti@unipr.it (S.B.); tiziana.dadda@unipr.it (T.D.); laura.giovati@unipr.it (L.G.); 2 Transfusion Medicine Unit, Azienda USL-IRCCS di Reggio Emilia, 42122 Reggio Emilia, Italy

**Keywords:** antifungal peptides, *Candida albicans*, circular dichroism spectroscopy, confocal microscopy, electron microscopy, *Galleria mellonella* model, self-assembly peptides, structure-function relationship

## Abstract

The previously described decapeptide AKVTMTCSAS (killer peptide, KP), derived from the variable region of a recombinant yeast killer toxin-like anti-idiotypic antibody, proved to exert a variety of antimicrobial, antiviral, and immunomodulatory activities. It also showed a peculiar self-assembly ability, likely responsible for the therapeutic effect in animal models of systemic and mucosal candidiasis. The present study analyzed the biological and structural properties of peptides derived from KP by substitution or deletion of the first residue, leaving unchanged the remaining amino acids. The investigated peptides proved to exert differential in vitro and/or in vivo anti-*Candida* activity without showing toxic effects on mammalian cells. The change of the first residue in KP amino acidic sequence affected the conformation of the resulting peptides in solution, as assessed by circular dichroism spectroscopy. KP-derivatives, except one, were able to induce apoptosis in yeast cells, like KP itself. ROS production and changes in mitochondrial transmembrane potential were also observed. Confocal and transmission electron microscopy studies allowed to establish that selected peptides could penetrate within *C. albicans* cells and cause gross morphological alterations. Overall, the physical and chemical properties of the first residue were found to be important for peptide conformation, candidacidal activity and possible mechanism of action. Small antimicrobial peptides could be exploited for the development of a new generation of antifungal drugs, given their relative low cost and ease of production as well as the possibility of devising novel delivery systems.

## 1. Introduction

In the last few decades, the attention of scientists, public health officials, governments, and general public has again focused on infectious diseases, due to emerging and re-emerging etiological agents, including multidrug-resistant pathogens [1,2,3,4]. Unfortunately, the spread of resistant microorganisms has not seen a simultaneous increase in the availability of new antimicrobials [5]. In this scenario, a number of alternative anti-infective strategies are being developed [6,7,8,9,10], including the exploitation of proteins and peptides as possible substitutes of conventional anti-infective drugs [11,12].

Over time, hundreds of natural small proteins and peptides produced by bacteria, fungi, plants, and animals (from the simplest sponges to mammals) have been characterized for their antimicrobial and/or antiviral activity. Semisynthetic, synthetic, and in silico predicted antimicrobial/antiviral peptides have been also described [13,14,15,16,17,18,19,20]. Bioactive peptides representing fragments of large parental proteins are called cryptides. Antimicrobial cryptides have been identified in common mammalian proteins, as hemoglobin, albumin, immunoglobulins, lactoferrin and salivary proteins, as well as in plant ribosome inactivating protein [21,22,23]. Our research group focused, in particular, on the anti-infective properties of synthetic peptides whose sequence represents fragments of variable and constant regions of antibodies (Ab) [24]. Several years ago, the work started from the decapeptide AKVTMTCSAS (A10S, killer peptide KP). KP, derived from the variable region of a recombinant anti-idiotypic antibody that functionally mimicked a yeast killer toxin, demonstrated a remarkable candidacidal activity in vitro and a therapeutic effect in murine models of mucosal and systemic candidiasis [25]. In further studies, KP proved to exert a significant activity against other important microbial and viral pathogens and showed immunomodulatory properties against dendritic cells (reviewed in [26]). Moreover, a peculiar self-assembly ability was demonstrated for KP, likely responsible for its therapeutic activity in vivo, that characterizes this peptide as a prototype of auto-delivering drugs [27].

The purpose of this work was to study the biological properties of KP-derivatives obtained by deleting the first residue (alanine) or replacing it with amino acids with different chemical-physical features. All the investigated KP-derivatives proved to be fungicidal in vitro against *Candida albicans*, chosen as reference microorganism, without showing toxic effects against mammalian cells. Some of them, however, unlike the parental peptide, did not exhibit a therapeutic effect in vivo in the experimental model of systemic candidiasis in *Galleria mellonella*.

Overall, the physical and chemical properties of the amino acid that occupies the N-terminal position in KP sequence influence the peptide conformation, candidacidal activity, and its possible mechanism of action. These findings provide valuable hints to enhance the biological activity and to optimize the stability of a recognized antimicrobial peptide.

## 2. Materials and Methods

### 2.1. Selection, Synthesis and Evaluation of the Hemolytic and Cytotoxic Activity of KP-Derivatives

The peptides investigated in this study derived from KP (AKVTMTCSAS) by deletion of the first non-polar amino acid alanine, or its replacement with histidine, lysine, leucine, proline, serine and tyrosine (two basic polar, two non-polar, and two polar amino acids, respectively) (Table 1). KP and its derivatives were synthesized as previously described [28], purified by HPLC, and their experimental molecular masses have been verified by mass spectrometry, at CRIBI-Peptide Facility (University of Padua, Padua, Italy). A stock solution of peptides (20 mg/mL) was prepared in dimethyl sulfoxide (DMSO), then proper dilutions were made for evaluation of biological activities. DMSO at proper concentration was present in controls (without peptides). All the peptides were evaluated for their hemolytic activity against freshly collected human red blood cells, while cytotoxicity against monkey kidney epithelial cells LLC-MK2 was determined by resazurin assay, as previously described [29].

### 2.2. Evaluation of the In Vitro Candidacidal Activity of KP-Derivatives

The candidacidal activity of KP-derivatives, in comparison to KP, was evaluated by a previously described colony forming unit (CFU) assay against the reference *C. albicans* strain SC5314 [25]. Briefly, approximately 500 yeast cells were incubated at 37 °C for 6 h in 100 µL of distilled water in the presence or absence (control growth) of the synthetic peptides at serial dilutions. After spreading on Sabouraud Dextrose agar (SDA) plates and incubation for 48–72 h at 30 °C, CFUs were counted. Peptide fungicidal activity was determined as percentage of CFU inhibition, according to the formula 100-(CFUs peptide treated/CFUs control growth) × 100. Each assay was carried out in triplicate and at least two independent experiments were performed for each condition. Half maximal effective concentration (EC_50_) was calculated by nonlinear regression analysis using GraphPad Prism 5 software (San Diego, CA, USA). CFU assays were also performed in order to verify if laminarin (100 or 200 µg/mL), a soluble β-1,3-glucan, could neutralize the candidacidal activity of KP-derivatives at their minimum fungicidal concentration [25]. Moreover, the kinetics of KP-derivatives killing activity at 2× the EC_50_ value was determined by CFU assays at 30, 60, 120, and 360 min. Based on the obtained EC_50_ values, the proper concentration of peptides to be used in subsequent biological assays was chosen taking into account the amount of yeast cells treated and the time of incubation. The candidacidal activity of the peptides (about 50% inhibition) under the adopted conditions was always confirmed by CFU assays.

### 2.3. Circular Dichroism (CD) Spectroscopy

CD measurements were performed on KP-derivatives on a Jasco 715 spectropolarimeter (JASCO International Co., Ltd, Tokyo, Japan) coupled to a Peltier PTC-348WI system for temperature control, according to previously adopted procedures [28]. Peptides (100 µM) were analyzed immediately after preparation of the starting aqueous solution (1 mM) and subsequently at different time points (3 and 24 months). The spectra of KP and its derivatives were also analyzed in presence of 500 µM laminarin.

### 2.4. Evaluation of In Vivo Toxicity and Therapeutic Activity of KP-Derivatives in G. mellonella

The *G. mellonella* model was adopted to evaluate in vivo toxicity and potential therapeutic effects of KP and its derivatives, as previously described [28]. Groups of sixteen larvae (400 ± 30 mg) at their final instar stage were used in all experiments. For preliminary evaluation of peptide toxicity larvae were inoculated with peptides (11 μmol/kg in 10 μL of saline) and scored daily for survival up to nine days. Groups of larvae untouched or inoculated with 10 μL of saline served as controls. For therapeutic evaluation, larvae were challenged with 5 × 10^5^ *C. albicans* cells/larva (in 10 μL saline) and injected after 30 min with the selected peptides (11 μmol/kg) or saline (control). After nine days monitoring, survival curves were compared through the Mantel–Cox log-rank test by Graph Pad Prism software. A value of *p* < 0.05 was considered significant.

### 2.5. Evaluation of Apoptosis Induction and Reactive Oxygen Species (ROS) Production in C. albicans after Treatment with KP-Derivatives

Induction of apoptosis in *C. albicans* SC5314 cells (5 × 10^5^ cells/mL) by treatment for 30 min with KP and its derivatives, at twice their EC_50_, was evaluated by the Muse Cell Analyzer (Merck Millipore, Darmstadt, Germany) using the Muse Annexin V & Dead Cell Assay kit, as previously described [28]. Data, acquired according to the manufacturer’s instructions, were reported as % of apoptotic cells (mean ± standard deviation from multiple experiments). Differences between peptide-treated groups and control (yeast cells in the absence of peptides) were assessed by Student’s *t* test. A value of *p* < 0.05 was considered significant.

Peptide-induced production of reactive oxygen species (ROS) in *C*. *albicans* SC5314 cells was evaluated by the 2′,7′-dichlorofluorescin diacetate (DCFH-DA) assay, according to a protocol previously described, with some modifications [30]. DCFH-DA is a cell-permeable non-fluorescent probe that turns to highly fluorescent DCFH upon oxidation. Yeast cells (2 × 10^7^ cells/mL) from a colony grown on SDA for 24 h at 37 °C were re-suspended in water (110 µL) in the presence or absence of 25 mM ascorbic acid and incubated for 30 min at room temperature (RT). Then KP and selected KP-derivatives were added at 20× the EC_50_ value in a final volume of 220 µL. As positive control, yeast cells were incubated in presence of caspofungin (20 µg/mL), an antifungal drug known for its ability to induce ROS production in *C. albicans* cells [31]. After incubation for 30 min at 37 °C, cells were centrifuged at 15,000× *g* for 10 min and pellets were re-suspended in PBS pH 7.4 with DCFH-DA (10 µg/mL) and incubated for 4 h at 37 °C in 96-well microplates (100 µL/well). Each assay was carried out in duplicate and at least two independent assays were performed for each condition. Fluorescence was measured every 60 min up to 4 h on a plate reader (EnSpire, PerkinElmer, Waltham, MA, USA) at excitation and emission wavelength of 485 and 540 nm, respectively.

### 2.6. Confocal Microscopy and Transmission Electron Microscopy (TEM) Studies

Confocal microscopy studies were performed by a LSM 510 Meta scan head integrated with the Axiovert 200 M inverted microscope (Carl Zeiss, Oberkochen, Germany) using selected peptides conjugated with fluorescein isothiocyanate (FITC), as previously described [28]. The interaction between labeled peptides and living *C. albicans* SC5314 cells were studied in time-lapse experiments. Yeast cells (2 × 10^7^/mL) were seeded (20 μL) on coverslips mounted in a special flow chamber and, after 30 min, the labeled peptide was added (final concentration 200 μM). Images were taken at 30 min intervals up to 6 h. Propidium iodide (PI) was added (1.5 μM) after 30 min. To evaluate changes in mitochondrial transmembrane potential (Δψ_m_), yeast cells in fresh YPD (yeast extract, peptone, dextrose) broth were loaded with 200 nM tetramethylrhodamine methyl ester (TMRM) for 1 h at RT in the dark. After centrifugation, yeast cells (20 μL) resuspended in 4% glucose with 200 nM TMRM, were seeded on coverslips and the baseline fluorescence was acquired. After 30 min, the labeled peptides were added (final concentration 200 μM), images were taken at different times and TMRM fluorescence intensity vs time calculated for the semi-quantitative analysis of Δψ_m_ variation. Each assay was carried out in triplicate.

TEM studies were performed on budding and germinating *C. albicans* SC5314 cells (7.5 × 10^8^ cells/mL), as previously described [32]. After 1 h incubation without (control) or with selected peptides (250 µM) yeast cells (50 μL volume) were pre-fixed for 5 min with 5% glutaraldehyde in 0.1 M PBS, pH 6.8. The suspensions were centrifuged at 5000× *g* for 5 min and cellular pellets were packed in 1% agarose and fixed with 2.5% glutaraldehyde in PBS for 3 h at RT, then left at 4 °C overnight. The agarose blocks were post-fixed for 30 min in 1% osmium tetroxide, then dehydration was performed by immersion in acetone gradient (25–100%). Infiltration in epoxy resin (Durcupan^TM^ ACM) was performed according to a standardized protocol [32]. Ultra-thin sections (80 nm) were contrasted with uranyl acetate (15 min) and lead citrate (5 min) then observed in a Philips EM 208S (Fei Europe, Eindhoven, The Netherlands) transmission electron microscope, operating at an accelerating voltage of 80 kV.

## 3. Results

### 3.1. In Vitro Fungicidal Activity, Hemolytic, and Cytotoxic Effects of KP-Derivatives

#### 3.1.1. Candidacidal Activity

The in vitro candidacidal activity of the investigated KP-derivatives, in comparison to the activity of KP, was evaluated against the reference *C. albicans* SC5314 strain by CFU assays. The obtained EC_50_ values are reported in Table 1. These values ranged between 0.28 and 1.34 µM. When compared to the parental KP peptide, H10S and K10S exhibited the highest activity, while L10S proved to be the less active.

The neutralization by laminarin, previously reported, suggested that the binding to β-glucans present in the yeast cell wall constitutes a critical step for KP candidacidal activity [25]. The results of CFU assays carried out, in the presence of laminarin, with KP-derivatives at their minimal fungicidal concentration are shown in Table 2. Candidacidal activity of H10S, K10S, L10S, and Y10S was almost or completely neutralized in the presence of laminarin at 100 µg/mL. A double concentration of laminarin (200 µg/mL) was needed for neutralization of KP, P10S, and K9S activity. In the latter condition, S10S retained a significant (>60%) candidacidal effect.

**Figure 1:**
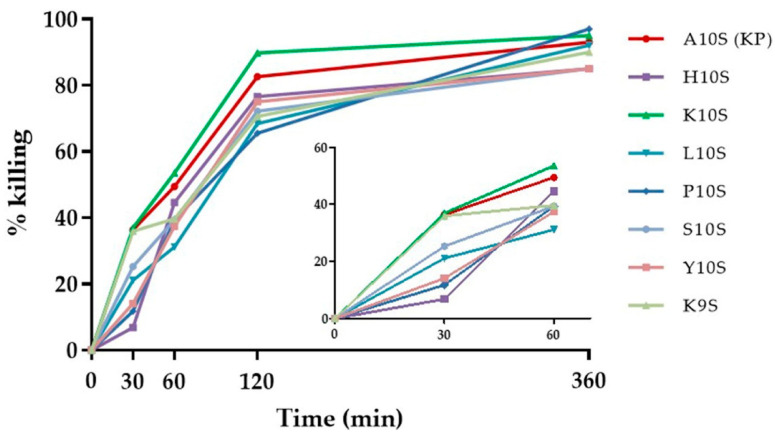
Time kinetics of *C. albicans* SC5314 killing. Peptides were used at their 2× EC_50_ value. The activity is expressed as percentage killing, calculated as: 100-(average number of CFUs in the peptide-treated group/average number of CFUs in the control group) × 100.

The rates of killing of *C. albicans* cells over time by KP and its derivatives were determined with peptides at twice their EC_50_ value (Figure 1). The two most active derivatives, K10S and H10S, showed a different initial behavior. K10S demonstrated a more rapid candidacidal effect, achieving 36.93% killing at 30 min, a value similar to the one obtained with the parental KP peptide (36.38%), while H10S attained only 6.85% killing.

#### 3.1.2. Hemolytic and Cytotoxic Activity

In comparison to negative and positive controls (0% hemolysis, PBS, and 100% hemolysis, Triton 1%, respectively), KP and its derivatives showed a negligible hemolytic activity at the tested concentrations (10, 25, and 50 µM). In fact, after 30 min incubation in the presence of the investigated peptides, lysed erythrocytes were always <1%, with the only exception of KP 25 µM (hemolysis 1.13%). After 120 min incubation, hemolysis was always <2%, with the only exception of K10S 25 µM (hemolysis 2.42%). Moreover, none of the investigated peptides showed a significant cytotoxicity against LLC-MK2 cells as assessed by the resazurin assay (Table S1). After 24 h incubation with peptides 10 µM, cell viability ranged between 93.25 and 100%, while with peptides 25 µM ranged between 90.20 and 100% (only exception for KP, viability 88.20%), and with peptides 50 µM ranged between 89.71 and 98.07%.

### 3.2. KP-Derivatives Conformational State

**Figure 2:**
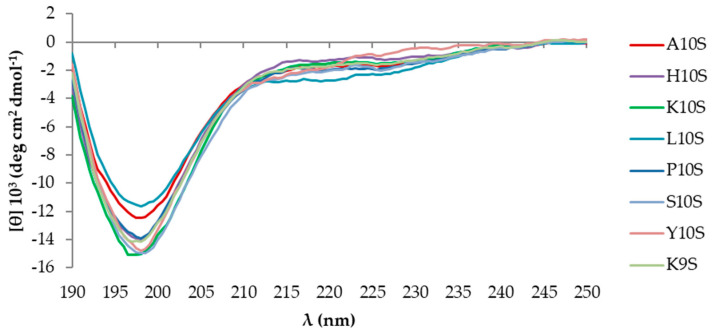
Far UV-CD spectra of 100 μM KP (A10S) and KP-derivatives acquired at 20 °C immediately after preparation of the starting aqueous solution (1 mM).

**Figure 3:**
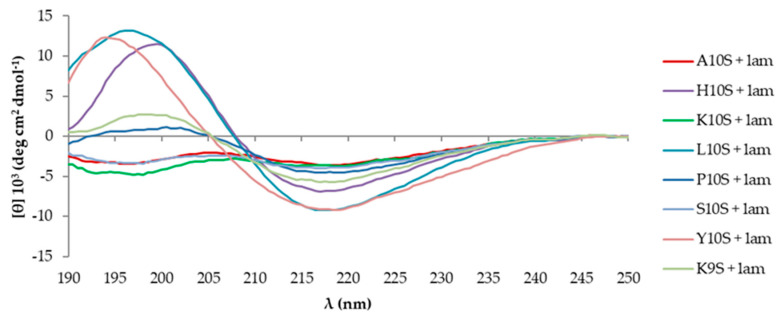
Far UV-CD spectra of 100 μM KP (A10S) and KP-derivatives acquired at 20 °C in the presence of laminarin (500 μM) immediately after preparation of the starting aqueous solution (1 mM).

Previous CD studies have revealed the ability of KP to acquire a β-sheet structure and spontaneously form large aggregates in aqueous solution over time, being this process strongly accelerated in the presence of laminarin or *C. albicans* cells [27]. To evaluate the possible contribution of each N-terminal residue to the conformational state of the investigated peptides, CD spectra of KP-derivatives were acquired soon after the solution in water (time 0) and after several months. At time 0 all the investigated peptides presented a typical random coil conformation, with a negative peak around 198 nm and a weak negative band at longer wavelengths (Figure 2). In the presence of laminarin, a change of the dichroic spectrum was observed for all peptides (Figure 3). After laminarin addition, the CD profile of K10S, the most active peptide, was characterized by a considerable decrease of the negative band at 198 nm and the appearance of a negative band at approximately 217 nm, as was for the parental peptide KP and S10S. The CD spectrum of H10S and L10S was characterized by a positive peak at 198 nm and a negative one at 217 nm, typical of β-sheet organized structures (Figure 3). Similar changes, although with some differences in band positions and intensities were recorded for Y10S, P10S, and K9S.

**Figure 4:**
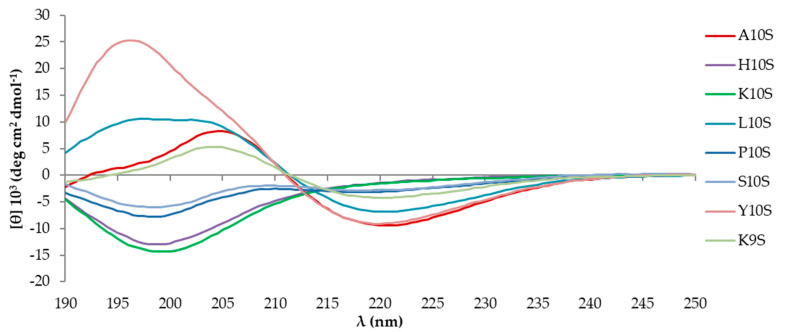
Far UV-CD spectra of 100 μM KP (A10S) and KP-derivatives acquired at 20 °C 24 months after preparation of the starting aqueous solution (1 mM).

Over time, in aqueous solution the peptides showed a differential behavior. Peptides KP, L10S, Y10S, and K9S acquired a CD profile with a negative band centered around 218 nm and a positive one in the range 198–205 nm, that suggests the acquisition of elements of β-sheet secondary structure mixed with a disordered component. On the other hand, H10S, K10S, P10S, and S10S were not able to spontaneously undergo any transition toward some sort of recognizable organized structure (Figure 4). Overall, the analysis of the CD spectra did not allow to extract any criteria that may correlate the presence of a specific amino acid in N-terminus with a particular conformational behavior of the peptide. It is clear, however, that each amino acid affects in its own way the spatial organization of the peptide in solution.

### 3.3. In Vivo Toxicity and Therapeutic Activity of KP-Derivatives in G. mellonella

**Figure 5:**
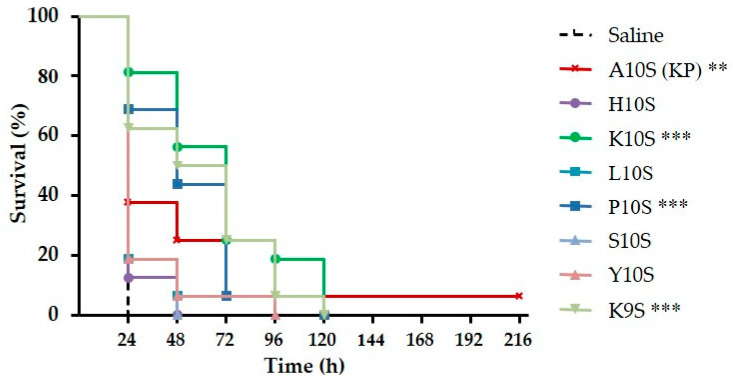
Therapeutic activity of KP-derivatives against experimental candidiasis in *Galleria mellonella*. Larvae were infected with 5 × 10^5^ cells of *C. albicans* SC5314 and treated with peptides (11 μmol/kg; single injection of 10 μL) or saline solution (control group). The survival curves of larvae treated with KP, K10S, P10S, and K9S were significantly different from that of the control group, as assessed by Mantel-Cox log rank test (** *p* < 0.01, *** *p* < 0.001).

Toxicity to *G. mellonella* larvae and therapeutic activity against experimental *C. albicans* infection were evaluated for all KP-derivatives in comparison with KP, whose therapeutic activity in consolidated models of murine systemic and mucosal candidiasis has been already described [25]. Under the adopted conditions, no significant difference in survival was observed between larvae inoculated with saline (control group), or with the peptides (11 μmol/kg), thus assessing their lack of toxicity in this experimental model (data not shown). After infection with a lethal inoculum of *C. albicans* SC5314 cells, a single injection (11 μmol/kg) of KP, K10S, P10S and K9S led to a significant increase in survival of larvae in comparison to infected animals inoculated with saline (Figure 5). Instead, H10S, L10S, S10S and Y10S did not show a therapeutic effect. Median survival time was 48, 60, and 72 h for larvae treated with P10S, K9S, and K10S, respectively, in comparison to 24 h for control group and groups treated with H10S, L10S, S10S, and Y10S. Median survival time of larvae treated with KP was 24 h, as for untreated controls. Nevertheless, 100% of untreated animals were dead 24 h post-infection, while survival of five KP-treated larvae was prolonged and 1/16 was still alive at day 9.

### 3.4. Apoptosis Induction and ROS Production in C. albicans Cells after Treatment with KP-Derivatives

**Figure 6:**
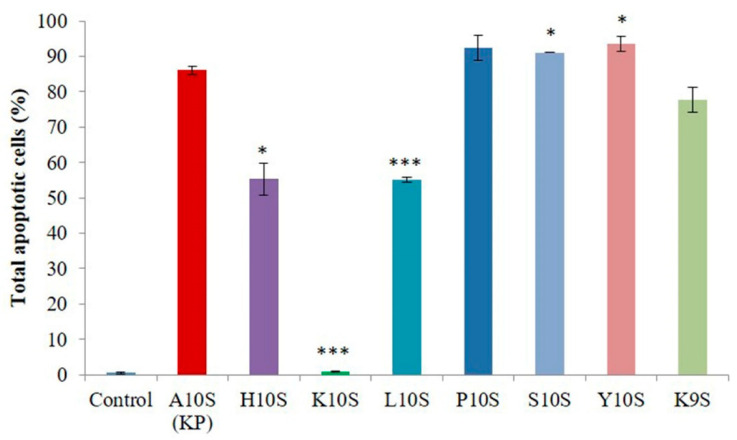
Apoptotic effects of treatment with KP and its derivatives in *Candida albicans* SC5314 cells. Yeast cells were treated for 30 min with peptides at 2× their EC_50_ values. Data, expressed as percentage of apoptotic cells on the total gated cells, represent the mean ± standard deviation from at least two independent experiments (* *p* < 0.05, *** *p* < 0.001 vs. KP-treated cells).

Flow cytometry was used to assess, through determination of phosphatidylserine externalization by reactivity with annexin V, if apoptosis is induced in *C. albicans* SC5314 whole cells after treatment with KP and its derivatives, at 2× their EC_50_ values. All investigated peptides were able to induce apoptosis, although to a different extent, except K10S (Figure 6). In fact, there was no statistically significant difference between the percentage of apoptotic cells induced by treatment with K10S and the one of the control (untreated cells). In comparison to the parental peptide KP, its derivatives showed a statistically significant improved (S10S and Y10S), decreased (H10S and L10S) or unchanged (P10S and K9S) ability to induce apoptosis in *C. albicans* cells. In particular, the in vitro most active peptides, H10S and K10S, showed a different behavior. While H10S maintained, although reduced, the apoptotic effect, K10S lost it completely. As an example, the apoptotic profile from a single assay performed on *C. albicans* cells treated with KP, H10S, and K10S is shown in Figure S1.

**Figure 7:**
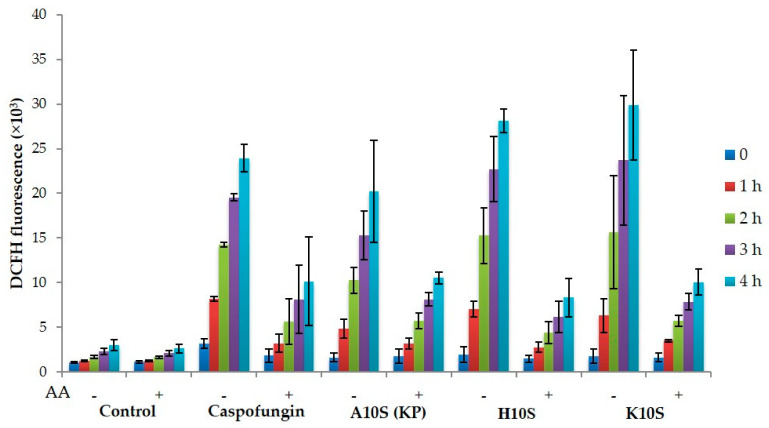
Intracellular ROS production induced by treatment with KP and selected KP-derivatives. After incubation with (+) or without (−) ascorbic acid (AA), *Candida albicans* SC5314 cells were treated for 30 min with caspofungin (CAS, 20 µg/mL, positive control) and peptides KP, H10S, and K10S (20× EC_50_), then DCFH-DA was added and fluorescence intensity was monitored at different times. Data represent the mean ± standard deviation from at least two independent experiments.

ROS production was evaluated in *C. albicans* cells after treatment with KP and its most active derivatives, H10S and K10S. The presence of ROS, revealed by a green fluorescence resulting from the oxidation of DCFH-DA into DCFH, was seen after treatment with all the tested peptides, in comparison to untreated control cells (Figure 7). Fluorescence intensity values were significantly lower in samples pre-incubated with ascorbic acid, a well-known antioxidant, for all time points, with the exception of KP-treatment at 60 and 240 min and K10S-treatment at 60 and 120 min.

### 3.5. Confocal Microscopy and Transmission Electron Microscopy (TEM) Studies on C. albicans Cells Treated with Selected KP-Derivatives

**Figure 8:**
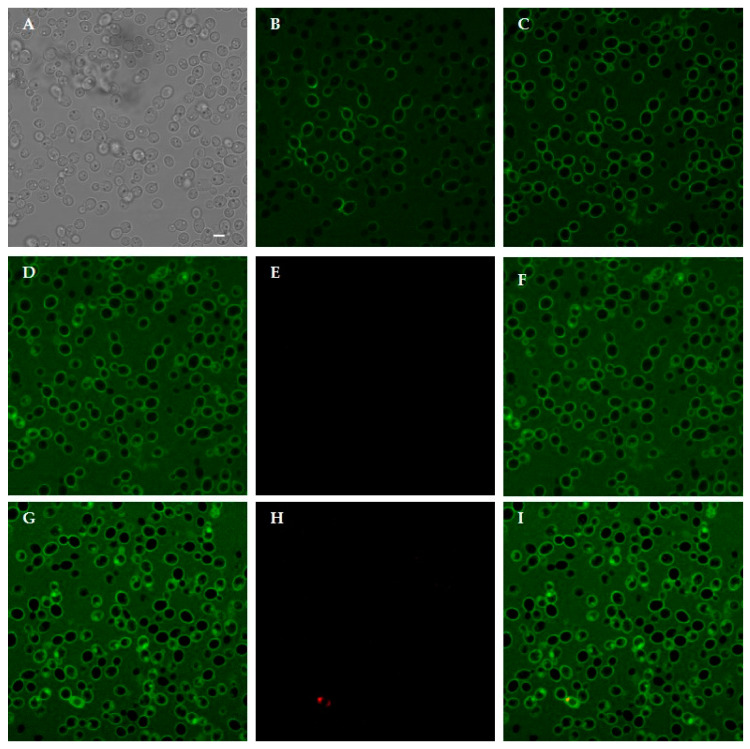
Interaction between living *Candida albicans* cells and FITC-labeled KP. Confocal microscopy images of yeast cells incubated for 10 min and 150 min with the labeled peptide are shown in (**B**,**C**), respectively. KP bound to yeast cell wall. In (**A**), the same field is shown in light transmission. Peptide internalization was observed after 240 min: (**D**) FITC; (**E**) PI; (**F**) merge of (**D**,**E**) and increased over time leading to cell death, as demonstrated by PI internalization after 360 min: (**G**) FITC; (**H**) PI; (**I**) merge of (**G**,**H**). Bar, 5 μm.

Time-lapse confocal microscopy allowed to investigate the dynamic interaction between selected KP-derivatives and living *C. albicans* cells. FITC-labeled A10S (KP), H10S and K10S proved to initially bind to yeast cell wall and enter living cells over time. This process, leading to cell death, as shown by PI internalization, took place at different times for the three peptides. As shown in Figure 8, FITC-labeled KP was detected mainly on the yeast cell wall up to 150 min (Figure 8B,C). After 240 min the peptide was detectable inside some yeast cells (Figure 8D) and an increase of the intracellular signal was observed over time (Figure 8G). After 360 min the first dead cells were seen (Figure 8H).

**Figure 9:**
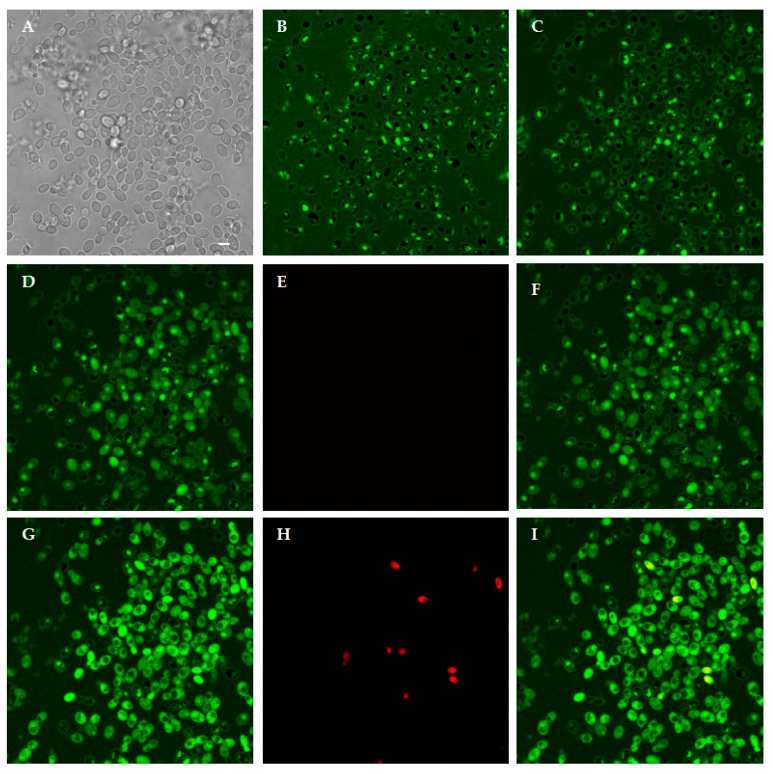
Interaction between living *Candida albicans* cells and FITC-labeled H10S. Confocal microscopy images of yeast cells incubated for 10 min and 180 min with the labeled peptide are shown in (**B**,**C**), respectively. H10S was internalized in most yeast cells. In (**A**), the same field is shown in light transmission. Peptide internalization increased in viable cells after 240 min: (**D**) FITC; (**E**) PI; (**F**) merge of (**D**,**E**), eventually leading to cell death, as demonstrated by PI internalization at 360 min: (**G**) FITC; (**H**) PI; (**I**) merge of (**G**,**H**). Bar, 5 μm.

Following treatment with FITC-labeled H10S (Figure 9), the peptide was already observed inside yeast cells after 10 min (Figure 9B). The intracellular signal progressively increased over time in still viable cells (Figure 9C, 180 min, Figure 9D, 240 min). After 360 min, some cells were completely fluorescent and no longer viable, as demonstrated by PI internalization.

**Figure 10:**
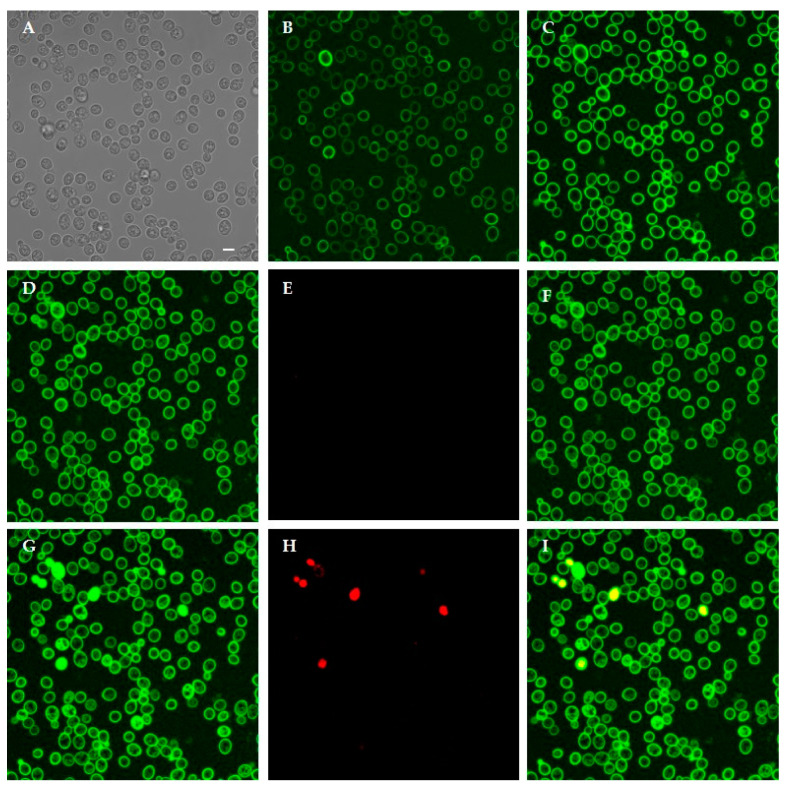
Interaction between living *Candida albicans* cells and FITC-labeled K10S. Confocal microscopy images of yeast cells incubated with the labeled peptide for 15 and 100 min are shown in (**B**,**C**), respectively. K10S was mainly bound to yeast cell wall. In (**A**), the same field is shown in light transmission. After 150 min, K10S entered viable yeast cells: (**D**) FITC; (**E**) PI; (**F**) merge of (**D**,**E**). Intensely fluorescent dead cells were observed after 180 min, as demonstrated by PI internalization: (**G**) FITC; (**H**) PI; (**I**) merge of (**G**,**H**). Bar, 5 μm.

Interaction of FITC-labeled K10S with yeast cells was similar to the one of the parental KP peptide, but with a faster kinetics. In fact, after initial binding to yeast cell walls, labeled peptide was detectable in most still viable yeast cells at 150 min, and after 180 min PI-positive dead cells were already detectable (Figure 10).

**Figure 11:**
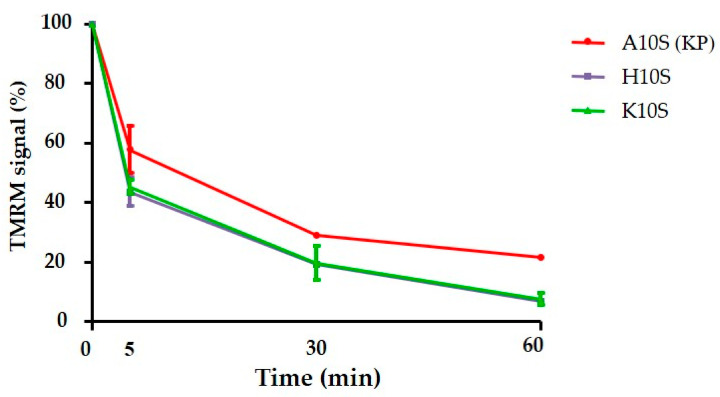
Change in TMRM fluorescence intensity in *Candida albicans* SC5314 cells after peptide treatment. Data represent the mean ± standard deviation from three independent experiments.

**Figure 12:**
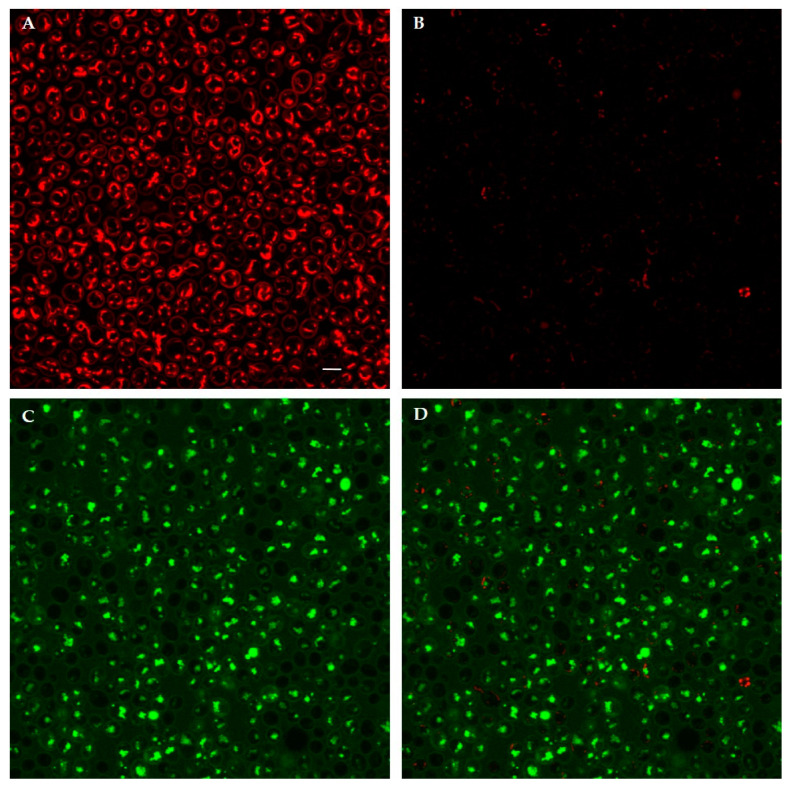
Confocal images of living *Candida albicans* SC5314 cells pre-loaded with TMRM then treated for 30 min with FITC-labeled H10S: (**B**) TMRM; (**C**) FITC; (**D**) merge of (**B**,**C**), in comparison with untreated (control) cells: (**A**) TMRM. Bar, 5 µm.

At last, confocal microscopy allowed a real-time visualization of changes in Δψ_m_ using TMRM as mitochondrial probe. TMRM was rapidly accumulated into mitochondria of living yeast cells. After addition of the FITC-labeled peptides, A10S (KP), H10S and K10S, a rapid decrease of TMRM signal was observed, starting from 5 min up to 60 min (Figure 11). Confocal images showed the general decrease of mitochondrial signal following peptide treatment. As an example, *C. albicans* cells loaded with TMRM and treated with FITC-labeled H10S are shown in Figure 12. In addition, confocal images allowed to observe the lack of co-localization between TMRM and FITC intracellular signals.

**Figure 13:**
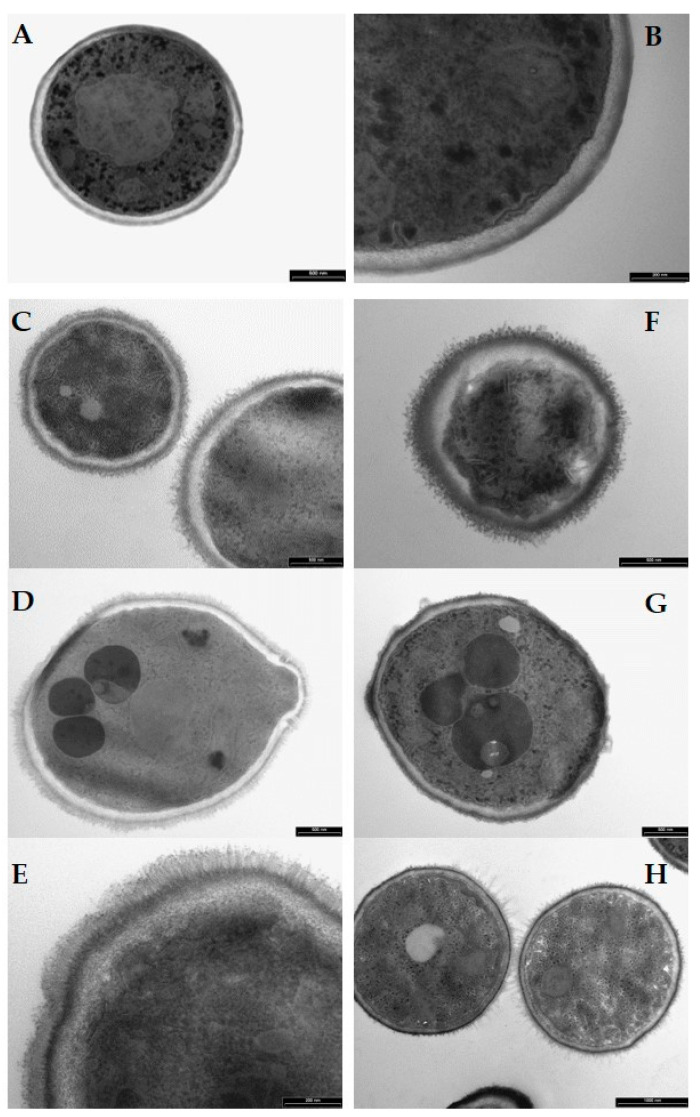
Transmission electron microscopy images of *Candida albicans* cells treated for 60 min with selected KP-derivatives. (**A**,**B**): untreated (control) cells. (**C**–**E**): H10S-treated cells. (**F**–**H**): K10S-treated cells. Yeast cells treated with H10S and K10S (250 µM) revealed structural and morphological alterations. Bars = 500 nm (**A**,**C**,**D**,**F**,**G**), 1000 nm (**H**), 200 nm (**B**,**E**).

TEM observation revealed morphological alterations in *C. albicans* cells treated with H10S and K10S peptides, similar to those previously observed for the parental peptide [33]. While untreated control cells showed a compact structure and a regular profile (Figure 13A,B), peptide-treated cells presented gross morphological alterations of intracellular structures, a cell wall swelling and a detachment of plasma membrane with numerous invaginations (Figure 13C, H10S-treated, and F, K10S-treated cells). In some yeast cells were present variable sized, dark round microbodies (Figure 13D, H10S-treated and G, K10S-treated cells). Moreover, peptide aggregates were observed on the surface of the treated cells (Figure 13C–E, H10S-treated cells; Figure 13F–H, K10S-treated cells).

## 4. Discussion

The need to develop new antimicrobial compounds with good pharmacokinetic and pharmacodynamic properties, devoid of toxic effects, and able to overcome resistance to conventional drugs is ever increasing. Among the potential candidate molecules, a great number of antimicrobial peptides from various sources has been described to date. In particular, KP has proven to possess extraordinary anti-infective and immunomodulatory activities, related to its structural properties. In fact, the peptide appears to be very stable and presents the ability to reversibly self-assemble in structures similar to hydrogels [26,27].

In order to better analyze and possibly optimize KP biological properties, some derivatives obtained by deletion or substitution of the N-terminal amino acid have been studied. None of the examined peptides proved to be significantly toxic in the adopted experimental conditions, even at high concentrations. All KP-derivatives proved to be candidacidal in vitro, showing EC_50_ values increased, decreased, or comparable to the one of the parental peptide.

In particular, the replacement of the first residue with polar amino acids positively charged at physiological pH, such as histidine (H10S) and lysine (K10S), greatly increased the fungicidal activity in vitro. These findings emphasize the role of the positive net charge of peptides in the interaction with the target microbial cells, critical to sustain the activity of antimicrobial peptides. However, this condition was not sufficient to ensure a therapeutic effect in vivo, since only the K10S peptide, in addition to P10S and K9S, was found protective in the experimental systemic candidiasis model in *G. mellonella*. It could be hypothesized that the lack of therapeutic activity in vivo of H10S relies on the initial slower kinetics of its candidacidal activity, as assessed in vitro (Figure 1) and/or on some sort of reduced stability of the peptide governed by the N-terminus residue.

The substitution of alanine with residues characterized by greater steric hindrance determined a decrease in candidacidal activity, as was the case with leucine (L10S) and, to a lesser extent, with tyrosine (Y10S). Possibly, this bulkiness impaired the organization in ordered β structures, as already inferred by the study of structure-function relationships of other antimicrobial antibody-derived peptides [28]. Instead, the structural rigidity of proline pyrrolidine ring at the N-terminal (P10S) did not change the candidacidal activity either in vitro or in vivo in comparison to the parental peptide.

**Figure 14:**
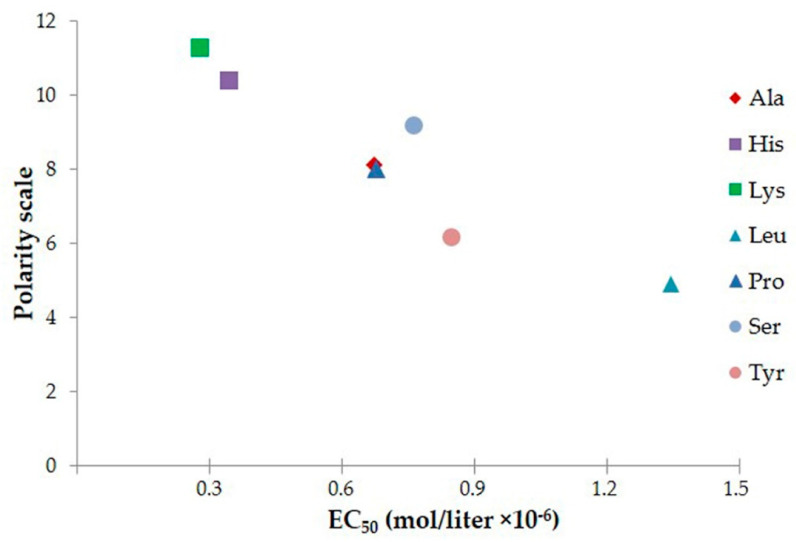
Correlation between polarity of the first residue and peptide EC_50_.

It was not possible to establish a common rule linking all involved amino acid properties, such as hydrophobicity, charge, polarity, steric arrangement. The polarity of the first residue, calculated according to amino acid scale values proposed by Grantham [34], seemed to be a good indicator of peptide candidacidal activity in vitro. In fact, for all considered peptides, EC_50_ values decreased with increasing polarity of the N-terminal amino acid (Figure 14).

It is known that the chemical and physical properties of a molecule are the result of a fine balance among various factors that encompass bond nature, stereo-chemical features, structural and dynamical properties. In the case of KP, the presence of a Cys residue and the alternation of hydrophobic and hydrophilic residues favor the possibility to acquire a β-sheet organization. All KP-derivatives studied in this work present a cysteine residue and a sequence with alternating hydrophobic and hydrophilic residues, nonetheless they show differences in the ability, or eventually inability, to acquire a β-sheet structure or any other well defined structural organization, thus rendering difficult to correlate the structure of the peptides with their biological function.

As already described for KP, the interaction with fungal cell wall structures, i.e., β-glucans, is an essential step for KP-derivatives action, as demonstrated through the neutralization of candidacidal activity by laminarin. Confocal microscopy involving H10S and K10S, the KP-derivatives characterized by the lower EC_50_ values, confirmed the initial binding on the yeast cell wall, while TEM images showed clearly visible peptide aggregates on yeast cell surface.

The kinetics of *Candida* killing suggested a non-membranolytic mechanism of action for KP and its derivatives. Most investigated peptides were capable of inducing apoptosis in yeast cells, as KP itself. On the contrary, K10S, the derivative characterized by the higher activity in vitro and a good therapeutic activity in vivo, did not induce apoptosis. K10S, as KP and H10S, induced the production of intracellular ROS and caused a rapid decrease in mitochondrial transmembrane potential. An increase of intracellular ROS without induction of apoptotic processes was already described after treatment of fungal cells with the salivary peptide histatin 5 and its derivative KM29 [35,36,37]. Further studies are needed to deepen knowledge on the exact mechanism of the candidacidal activity of the described peptides.

It is worth underlining that the only difference between KP and its derivatives is the N-terminus residue and that the major functional differences are observed in vivo. These observations remind the “N-terminus rule” [38] that attributes to the nature of the amino acid in N-terminus the capability to modulate the protein stability and therefore its functionality. Although this was referred to intracellular half-life of proteins, something similar could also apply to small peptides.

In conclusion, the peculiar properties of KP and its derivatives described in this study could establish them as interesting candidates to develop novel antifungal drugs, either for topical applications against mucosal candidiasis or even for systemic administration if a sufficient stability under physiological conditions could be verified.

## Figures and Tables

**Table 1:** In vitro fungicidal activity of KP and its derivatives against *Candida albicans* SC5314.

Peptide	Sequence	MM ^1^ (Da)	EC_50_^2^ (95 % Confidence Intervals) [mol/liter] × 10^−6^	EC_50_ Derivatives/EC_50_ KP
A10S (KP)	AKVTMTCSAS	998.17	0.673 (0.633–0.716)	-
H10S	HKVTMTCSAS	1064.21	0.343 (0.331–0.357)	0.51
K10S	KKVTMTCSAS	1055.26	0.277 (0.274–0.279)	0.41
L10S	LKVTMTCSAS	1040.25	1.344 (1.246–1.450)	2.00
P10S	PKVTMTCSAS	1024.21	0.676 (0.615–0.742)	1.00
S10S	SKVTMTCSAS	1014.17	0.761 (0.587–0.986)	1.13
Y10S	YKVTMTCSAS	1090.26	0.847 (0.766–0.936)	1.26
K9S	KVTMTCSAS	927.09	0.863 (0.653–1.141)	1.28

^1^ MM, molecular mass (dalton) calculated by the ExPASy tool ProtParam; ^2^ EC_50__,_ half maximal effective concentration, expressed in mol/liter × 10^−6^. In parentheses, 95% confidence intervals.

**Table 2:** Neutralization of in vitro candidacidal activity of KP and KP-derivatives by laminarin.

		*C. albicans* Growth Inhibition (%) in the Presence of	
Peptide	µg/mL ^1^	Laminarin 100 µg/mL	Laminarin 200 µg/mL
A10S (KP)	5.00	96.39 ± 1.68	1.80 ± 0.49
H10S	2.00	6.20 ± 3.71	n.d.
K10S	1.25	1.07 ± 0.39	n.d.
L10S	5.00	0	n.d.
P10S	5.00	97.71 ± 0.46	3.10 ± 4.38
S10S	5.00	99.12 ± 0.50	60.73 ± 13.13
Y10S	5.00	5.57 ± 4.84	n.d.
K9S	5.00	31.09 ± 1.71	0.68 ± 0.97

^1^ Each peptide was tested at its minimal candidacidal concentration, i.e., the concentration yielding no colonies (100% inhibition) in the CFU assay performed as described in Material and Methods section. n.d. = not determined.

## Data Availability

The data presented in this study are available in article and supplementary material.

## Supplementary Materials

The following are available online at https://www.mdpi.com/2309-608X/7/2/129/s1, Figure S1: Apoptotic profile of *Candida albicans* cells treated with KP and selected derivatives. Table S1: In vitro cytotoxic activity of KP and its derivatives against LLC-MK2 cells.
