# Supplementary material for: In Vitro and In Vivo Anti-Candida Activity and Structural Analysis of Killer Peptide (KP)-Derivatives
Source: J Fungi (Basel). 2021 Feb 10;7(2):129. doi: 10.3390/jof7020129 (PMC7916522; doi:10.3390/jof7020129)
Supplement: Supplementary file 1 [file jof-07-00129-s001.pdf]

Journal of *Fungi*

MDPI

In vitro and *in vivo* anti-*Candida* activity and structural analysis of killer peptide (KP)-derivatives

Tecla Ciociola [1], Thelma A. Pertinhez [1,2], Tiziano De Simone [1], Walter Magliani [1], Elena Ferrari [1], Silvana Belletti[1], Tiziana D'Adda [1], Stefania Conti [1,*] and Laura Giovati [1]

1 Department of Medicine and Surgery, University of Parma, Parma 43126, Italy; tecla.ciociola@unipr.it (T.C.); thelma.pertinhez@unipr.it (T.A.P.); tiziano.desimone@unipr.it (T.D.S.); walter.magliani@unipr.it (W.M.); elena.ferrari@unipr.it (E.F.); silvana.belletti@unipr.it (S.B.); tiziana.dadda@unipr.it (T.D.); laura.giovati@unipr.it (L.G.)

2 Transfusion Medicine Unit, Azienda USL-IRCCS di Reggio Emilia, Reggio Emilia 42122, Italy

* Correspondence: stefania.conti@unipr.it

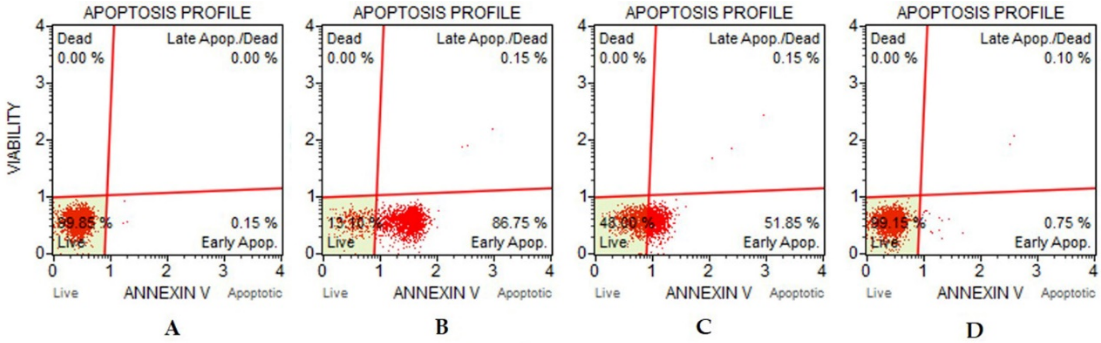

	A Cell Conc. (Cells / mL)	A % Gated	B Cell Conc. (Cells / mL)	B % Gated	C Cell Conc. (Cells / mL)	C % Gated	D Cell Conc. (Cells / mL)	D % Gated
Live (LL)	4.26E+05	99.85 %	6.10E+04	13.10 %	2.03E+05	48.00 %	4.12E+05	99.15 %
Early Apoptotic (LR)	6.40E+02	0.15 %	4.04E+05	86.75 %	2.20E+05	51.85 %	3.12E+03	0.75 %
Late Apop./ Dead (UR)	0.00E+00	0.00 %	6.99E+02	0.15 %	6.35E+02	0.15 %	4.15E+02	0.10 %
Debris (UL)	0.00E+00	0.00 %	0.00E+00	0.00 %	0.00E+00	0.00 %	0.00E+00	0.00 %
Total Apoptotic	6.40E+02	0.15 %	4.05E+05	86.90 %	2.20E+05	52.00 %	3.53E+03	0.85 %

Figure S1. Apoptotic profile of *Candida albicans* cells treated with KP and selected derivatives. (**A**): untreated control cells. (**B**): KP-treated cells. (**C**): H10S-treated cells. **D**: K10S-treated cells. Yeast cells (5×10^5 cells/ml) were treated for 30 minutes with peptides at 2× their EC_{50} values. LL: lower left quadrant; LR: lower right quadrant; UR: upper right quadrant; UL: upper left quadrant. Apoptotic profile was evaluated by the Muse Cell Analyzer (Merck Millipore) using the Muse Annexin V & Dead Cell Assay kit. Reported data derived from a single representative experiment.

Table S1. In vitro cytotoxic activity of KP and its derivatives against LLC-MK2 cells.

	Cell viability (%)		
**Peptide**	**10 µM**	**25 µM**	**50 µM**
A10S (KP)	93.59	88.20	89.71
H10S	96.77	94.90	90.99
K10S	93.74	100	90.95
L10S	95.70	93.44	98.07
P10S	93.25	90.20	89.77
S10S	99.77	99.75	91.09
Y10S	96.89	95.35	97.18
K9S	100	98.55	95.86
